# Molecular Epidemiology of Foot-and-Mouth Disease Viruses in the Emirate of Abu Dhabi, United Arab Emirates

**DOI:** 10.3390/vetsci11010032

**Published:** 2024-01-15

**Authors:** Yassir M. Eltahir, Hassan Zackaria Ali Ishag, Jemma Wadsworth, Hayley M. Hicks, Nick J. Knowles, Valérie Mioulet, Donald P. King, Meera Saeed Mohamed, Oum Keltoum Bensalah, Mohd Farouk Yusof, Esmat Faisal Malik Gasim, Zulaikha Mohamed Al Hammadi, Asma Abdi Mohamed Shah, Yasir Ali Abdelmagid, Moustafa Abdel meguid El Gahlan, Mohanned Fawzi Kassim, Kaltham Kayaf, Ahmed Zahran, Mervat Mari Al Nuaimat

**Affiliations:** 1 Animals Extension and Health Services Division, Abu Dhabi Agriculture and Food Safety Authority (ADAFSA), Abu Dhabi P.O. Box 52150, United Arab Emirates; 2 Biosecurity Affairs Division, Development & Innovation Sector, Abu Dhabi Agriculture and Food Safety Authority (ADAFSA), Abu Dhabi P.O. Box 52150, United Arab Emirates; 3 FAO World Reference Laboratory for FMD (WRLFMD), The Pirbright Institute, Pirbright GU24, UK; 4 Animal Development & Health Department, Ministry of Climate Change & Environment, Dubai P.O. Box 1509, United Arab Emirate

**Keywords:** foot-and-mouth disease virus, United Arab Emirates, epidemiology, VP1, sequencing, phylogenetic, prototype

## Abstract

**Simple Summary:**

Foot-and-mouth disease (FMD) is endemic in the United Arab Emirates (UAE); however, FMD virus (FMDV) genotyping in small ruminants or cattle has never been reported. This study focused on FMD outbreaks that occurred during 2021 in two different regions in the Emirate of Abu Dhabi, where cases affected sheep, goats, cattle, and *Arabian oryx*. VP1 sequences from different isolates were characterised, and phylogenetic analysis revealed epidemiological connections between FMDV sequences collected in the UAE and neighbouring countries, which highlights the importance of implementing measures such as vaccination, animal movement controls, and biosecurity to limit the spread of the disease.

**Abstract:**

Foot-and-mouth disease (FMD) is an endemic disease in the United Arab Emirates (UAE) in both wild and domestic animals. Despite this, no systematic FMD outbreak investigation accompanied by molecular characterisation of FMD viruses (FMDVs) in small ruminants or cattle has been performed, and only a single report that describes sequences for FMDVs in wildlife from the Emirate has been published. In this study, FMD outbreaks that occurred in 2021 in five animal farms and one animal market in the Emirate of Abu Dhabi were investigated. Cases involved sheep, goats, and cattle, as well as *Arabian oryx* (*Oryx leucoryx*). Twelve samples were positive for FMDV via RT-qPCR, and four samples (*Arabian oryx n* = 1, goat *n* = 2, and sheep *n* = 1) were successfully genotyped using VP1 nucleotide sequencing. These sequences shared 88~98% identity and were classified within the serotype O, Middle East–South Asia topotype (O/ME-SA). Phylogenetic analysis revealed that the *Arabian oryx* isolate (UAE/2/2021) belonged to the PanAsia-2 lineage, the ANT-10 sublineage, and was closely related to the FMDVs recently detected in neighbouring countries. The FMDV isolates from goats (UAE/10/2021 and UAE/11/2021) and from sheep (UAE/14/2021) formed a monophyletic cluster within the SA-2018 lineage that contained viruses from Bangladesh, India, and Sri Lanka. This is the first study describing the circulation of the FMDV O/ME-SA/SA-2018 sublineage in the UAE. These data shed light on the epidemiology of FMD in the UAE and motivate further systematic epidemiological studies and genomic sequencing to enhance the ongoing national animal health FMD control plan.

## 1. Introduction

Foot-and-mouth disease (FMD), a highly contagious transboundary viral disease affecting all cloven-hoofed animals, is considered a major worldwide constraint to animal production and international trade [1]. The disease affects approximately 77% of global livestock population, particularly in Africa, the Middle East, and Asia [2]. FMD is caused by the FMD virus (FMDV), genus Aphthovirus, family Picornaviridae [3]. The viral genome consists of 8500 base pairs (bps), and it contains an open reading frame (ORF) flanked by 5′ and 3′-untranslated regions (UTR). This ORF (a polyprotein of 2300 amino acids) is further processed by viral proteases, resulting in the formation of mature viral proteins and precursors. Four structural (VP1, VP2, VP3, and VP4) and ten nonstructural proteins are produced during this process [4].

FMDV induces vesicles on the feet, mammary glands, and oral cavity in the infected animal [3]. It causes weight loss and significant declines of milk production in dairy animals [5]. The disease may cause high mortality in young animals due to cardiac arrest succeeding myocarditis [6]. In a fully susceptible livestock population, the morbidity rate of FMDV can be as high as 100% with a high mortality rate. However, the morbidity and mortality rates of FMDV depend on various factors, such as the animal species, breed, production type, age, immunity, virus dose, and animal movement [7]. FMD can persist in goats and sheep for up to nine months [8]. The FMD is diagnosed using a combination of history, clinical symptoms, and laboratory tests. FMDV can be isolated in cell cultures, viral nonstructural proteins can be detected using enzyme-linked immunosorbent assays (ELISAs), and viral genomic material can be detected using polymerase chain reaction (PCR) assays [9]. Anti-nonstructural protein (NSP) antibody testing is commonly used to differentiate infected animals from vaccinated animals in FMD endemic areas [10] and FMD-free countries [11]. FMD outbreaks are widespread in market-oriented systems compared to subsistence systems due to frequent movement and mixing of animals [12]. FMDV can be transmitted directly via inhalation of virus particles through direct contact with the acutely infected animals [13] or indirectly via a contaminated environment, as the virus can survive for a long period under favourable conditions [14,15], such as temperatures <50 °C, relative humidity >55%, and neutral pH [6,7,8]. Airborne transmission has also been reported over both long and short distances [16,17].

FMDVs comprise seven immunologically distinct serotypes (O, A, C, Asia 1, SAT 1, SAT 2, and SAT 3), although one of them, serotype C, is considered eradicated. More than 60 subtypes with variable antigens and different degrees of virulence have been found without cross-immunity between serotypes, and this causes difficulties in selecting the suitable vaccinal strains and/or subtypes contents to be used in vaccination and control programs adopted in a specific country or region [18,19]. Molecular epidemiology studies of FMDV based on analyses of the genomic sequences encoding one of the capsid proteins (VP1) are utilised to classify field strains, monitor virus outbreaks, and trace transboundary movements of virus lineages, as well as in the development and assessment of effective control strategies in a specific country or region [20]. Seven virus pools (1–7) have been proposed to define the geographical circulation of the seven FMDV serotypes [21,22,23]. The United Arab Emirates (UAE) is located on the Arabian Peninsula within Pool 3, which is home to serotypes O, A, and Asia 1. The principal topotypes/lineages currently occurring in this pool are O/ME-SA/PanAsia-2, A/ASIA/Iran-05, and Asia 1/ASIA/Sindh-08 [21,24], but the region has also recently experienced incursions of O/ME-SA/Ind-2001 and A/ASIA/G-VII from Pool 2 (South Asia) and SAT 2/XIV from Pool 4 (East Africa) [25,26,27].

The total animal population in the UAE is approximately 5 million, comprising 4.35 million small ruminants, 550,000 camels, and 110,000 cattle. FMD is a notifiable disease in the UAE, and since the first official report to the World Animal Information System (WAHIS) in 2003, FMD has been reported regularly due to cases affecting domesticated animals and wildlife. Up to 2023, a total number of 28 FMD outbreaks were reported to WAHIS [28]. Although FMD is recognised to be present, the epidemiology and evolution of FMDV strains circulating in UAE are poorly understood, and only a single paper that focused on FMD cases in captive scimitar-horned oryx (*Oryx dammah*) has described genetic lineages of FMDV that are circulating in the Abu Dhabi Emirate [29]. Understanding the local situation of FMD within the UAE and the identification of the risk factors responsible for FMDV occurrence and spread, in addition to knowledge of FMDV filed circulating subtypes, is a crucial factor for effective FMD control. This, in turn, provides useful information about emerging FMDV strains and continuous monitoring of disease outbreaks, supporting FMD control strategies in the region [30].

In order to address gaps in the molecular characterisation of FMDV in small ruminants and cattle, this study characterised FMDVs recovered during 2021 from goats, sheep, and *Arabian oryx* (*Oryx leucoryx*) in the Emirate of Abu Dhabi. The VP1 of the FMDVs causing these outbreaks was characterised and phylogenetically analysed. The results of the study provide crucial information about the epidemiology of FMD in the UAE and encourage continuous monitoring and further genetic characterisation of the circulating FMDVs to support FMD control strategies in the region. Further large-scale epidemiological studies are required, which would help prevent the spread of FMDV in the region and contribute to the FMD Global Control Strategy.

## 2. Materials and Methods

### 2.1. Outbreaks Investigations

**Figure 1:**
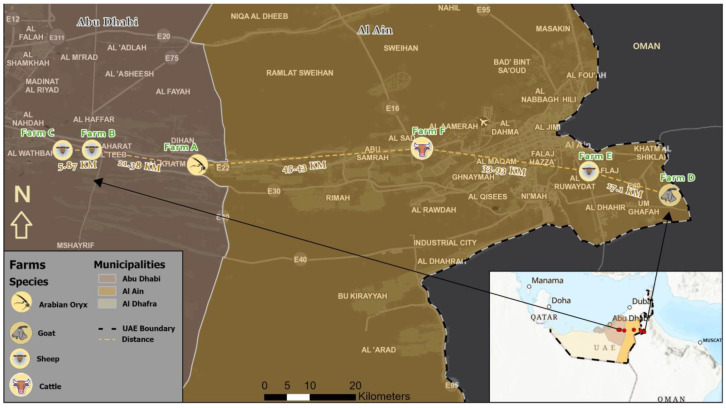
Map showing the location of the FMD-infected farms in the Abu Dhabi region (Farms A–C) and Al Ain region (Farms D–F), Abu Dhabi, UAE.

Outbreaks investigations included *Arabian oryx*, sheep, goats, and cattle located on five different farms and a livestock market. Clinical signs in the affected animals included pyrexia, lameness, and vesicular lesions. Three of the farms (A–C) were in the Abu Dhabi region, while two farms (D and E) and the animal market (F) were in the Al Ain region of the Abu Dhabi Emirate (Figure 1). 

The highest morbidity (6.8%) and mortality (1.7%) rates were observed in sheep (farm B) and in sheep and goats (farm D) located in the Abu Dhabi and Al Ain regions, respectively. The highest case fatality rate (25%) was observed on farms (A, C, and E) where FMD cases affected *Arabian oryx* and sheep.

The infected farms B, D, and E, containing sheep and goats, were not vaccinated against FMD. Similarly, the animals infected at the market (F) were not vaccinated. Farm A, which contained vaccinated sheep and goats, was subjected to an introduction of infected unvaccinated *Arabian oryx* three weeks before the outbreak from a captive facility within the Abu Dhabi Emirate, where an FMD outbreak had been recently observed. Introduction of unvaccinated goats was also reported on farm D. Farm C, which received a single dose of FMD vaccine two months before the outbreak, was located within a 5 Km radius of the infected farm B (Table 1).

### 2.2. Clinical Samples 

A total of 12 clinical swab samples (*Arabian oryx n* = 1, goats *n* = 2, sheep *n* = 6, and cattle *n* = 3) were collected from the farms and market and submitted to ADAFSA Veterinary Laboratories for FMD diagnosis via RT-qPCR (described below in Table 2). The FMDV-positive samples (*n* = 12) were dispatched under dry ice as dangerous biological substance category B UN 3373 to the FAO World Reference Laboratory for Foot-and-Mouth Disease (WRLFMD) (Pirbright Institute, Surrey, UK). Samples were kept refrigerated until reaching the laboratory, where they were kept at −20 °C for further studies, including virus genotyping and phylogenetic analysis. 

### 2.3. Detection of FMDV via Real-Time Quantitative PCR (RT-qPCR) and Serotyping

Total RNA was extracted from each mouth swab using the EZ1 Virus Mini Kit V2.0 (48) (Qiagen, Hilden, Germany) and Advanced EZ1 instrument (Qiagen in Hilden, Germany) as per the instructions. The total volume of sample loaded into the machine was 400 µL, and a total elution volume of 60 µL was collected. The presence of FMDV RNA was detected via RT-qPCR, as previously described [31]. The primers used were Callahan 3DF forward primer (5′-ACTGGGTTTTACAAACCTGTGA-3′) and Callahan 3DR reverse primer (5′-GCGAGTCCTGCCACGGA-3′), and the probe was Callahan 3D probe (FAM-5′-CTTCCTTTGCA CGCCGTGGGAC-3′-TAMRA). The PCR master mix (Real-Time ready RNA Virus Master Kit, Roche, Basel, Switzerland) was prepared in a total volume of 15 µL. It consisted of 7.6 µL of water, 4 µL of reaction buffer (5×), 0.4 µL of enzyme mix (50 ×), and 1 µL of each primer (10 pmol/µL) and probe (10 pmol/µL). Template RNA was added at 5 µL for a complete volume of 20 µL. The mixture was placed in BioRad CFX 96 with the following cycling conditions: reverse transcription (58 °C for 8 min), enzyme activation and/or initial denaturation (95 °C for 30 s), and 50 cycles of amplification (95 °C for 15 s and 60 °C for 1 min). 

At the WRLFMD, samples were tested via RT-qPCR [31,32], and virus isolation was performed using WRL-LFBK cells, as previously described [33,34]. Samples were reported FMDV-positive and submitted for genotyping if a cytopathogenic effect (CPE) was observed within 48 h of incubation of the cells at 37 °C. 

### 2.4. Sanger Sequencing and Phylogenetic Analysis

For this study, VP1 sequences were determined at WRLFMD using the previously described method [20]. Briefly, for each serotype, two independent RT-PCR assays were performed using the following primer pairs: O-1C244F/EUR-2B52R (type O) and FMD-3161F/FMD-4303R (FMDV universal). Sanger sequencing was performed on an ABI 3730xl DNA Analyzer (ABI Biosystems, Waltham, Massachusetts). 

The assembled VP1 sequences of UAE-FMDVs obtained in this study, along with other UAE-FMD strains of the O serotype retrieved from the NCBI GenBank database and the corresponding VP1 sequences (O serotype) downloaded from WRLFMD (FMD prototype strains from World Reference Laboratory for Foot-and-Mouth Disease (wrlfmd.org (accessed on 02 March 2023)), were multiple-aligned with ClustalW [35], available in the MEGA 11 program [36], to characterise the virus into its serotype and topotype as well as the prototype. The phylogenetic tree was constructed with MEGA 11 using the Maximum Likelihood method with 1000 Bootstrap confidence. The nucleotide sequence identity of VP1 sequences was also compared.

## 3. Results

### 3.1. FMD-Positive Samples

All 12 collected samples from farms (A-F) tested positive for FMDV using RT-qPCR at ADAFSA veterinary laboratories. At the WRLFMD, four of these samples showed CPE within 24–48 h of being inoculated onto LFBK αVβ6 cells and were subjected to VP1 nucleotide sequencing, comprising one from *Arabian oryx* (UAE/2/2021), two from goats (UAE/10/2021 and UAE/11/2021), and one from sheep (UAE/14/2021). The characterised VP1 sequences were submitted to GenBank and received the accession numbers OR425052, OR425054, OR425055, and OR425056 for O/UAE/2/2021, O/UAE/10/2021, O/UAE/11/2021, and O/UAE/14/2021, respectively. Samples from cattle were not successfully genotyped at WRLFMD.

### 3.2. Phylogenetic Analysis

**Figure 2:**
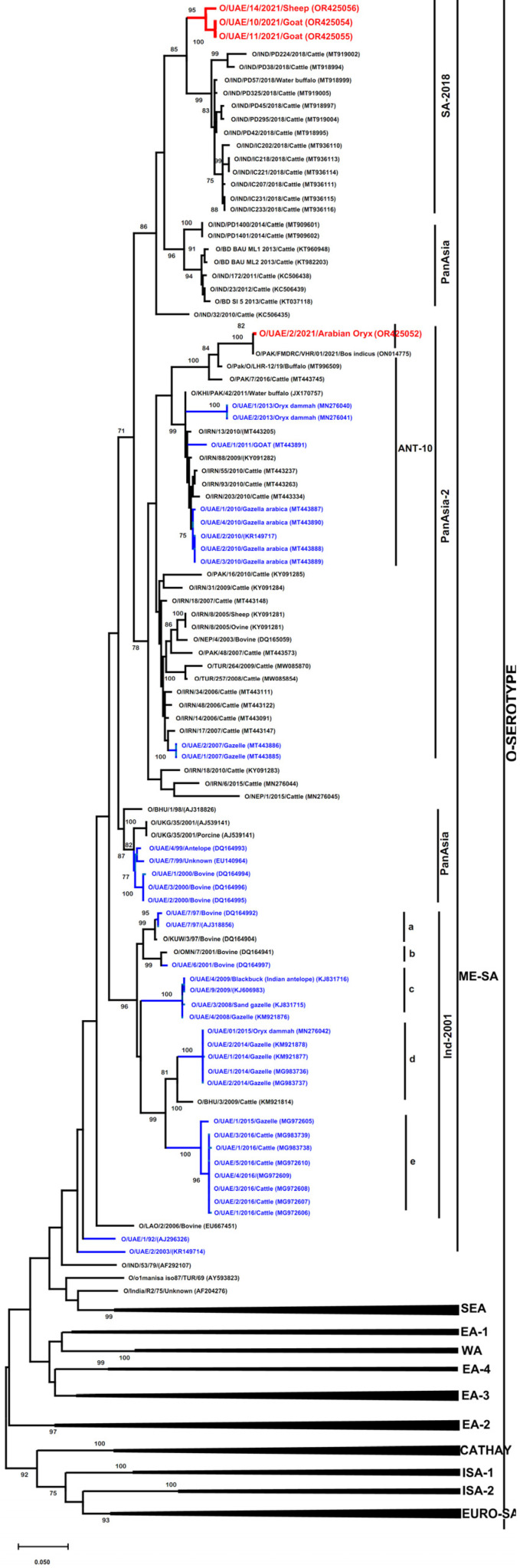
VP1-coding region phylogenetic relationship between FMDVs detected in the UAE in goats, sheep, and *Arabian oryx* and other FMDV sequences (in total, 135 sequences). Alignments were calculated with ClustalW implemented in MEGA 11. The tree was constructed using the Maximum Likelihood method in MEGA 11. Bootstrapping was performed with 1000 replicates and the value was indicated (bootstrap values of 70% and above are shown). The UAE sequences obtained in this study are denoted in red and are marked with red symbols. Previous FMDV VP1 sequences from UAE are shown with blue text.

The four VP1 sequences were subjected to phylogenetic analysis which showed that they all belonged to the O serotype/ME-SA topotype. The sequence for UAE/2/2021 belonged to the O/ME-SA/PanAsia-2 lineage/ANT-10 sublineage, where it clustered with FMDV sequences previously reported from cattle and water buffalo from Pakistan, cattle from Iran, and gazelle and goats from the UAE. The remaining three samples from goats and sheep (UAE/10/2021, UAE/11/2021, and UAE/14/2021) belonged to the O/ME-SA/SA-2018 lineage. They clustered distantly from other serotype O FMD lineages (Ind-2001, PanAsia, and PanAsia-2) previously reported in cattle and *Arabian oryx* from the UAE (Figure 2). Within lineage O/ME-SA/SA-2018, the UAE-FMD isolates formed a monophyletic group with a high bootstrap value, separated from sequences for other isolates originating from cattle and water buffalo from India and Sri Lanka. 

The VP1 sequences of UAE/2/2021 isolated from the *Arabian oryx* in farm A were most closely related to isolate [O/Pak/FMDRC/VHR/01/2021/Bos indicus (ON014775)], which was recently detected in cattle in Pakistan in 2021 and was more distantly separated, with 89–95% nucleotide identity from other FMD viruses detected in buffalo, cattle, *Oryx dammah*, or goats in Pakistan, Iran, or the UAE. 

The VP1 sequences of UAE/10/2021 and UAE/11/2021 isolated from goats from farm D were 100% identical to each other. The UAE sequences which originated from two different farms (D and E) shared 98% identity between them and were more distantly separated, with 93% to 95% nucleotide identities compared to the VP1 sequences detected in cattle and water buffalo from India and Sri Lanka, respectively.

**Figure 3:**
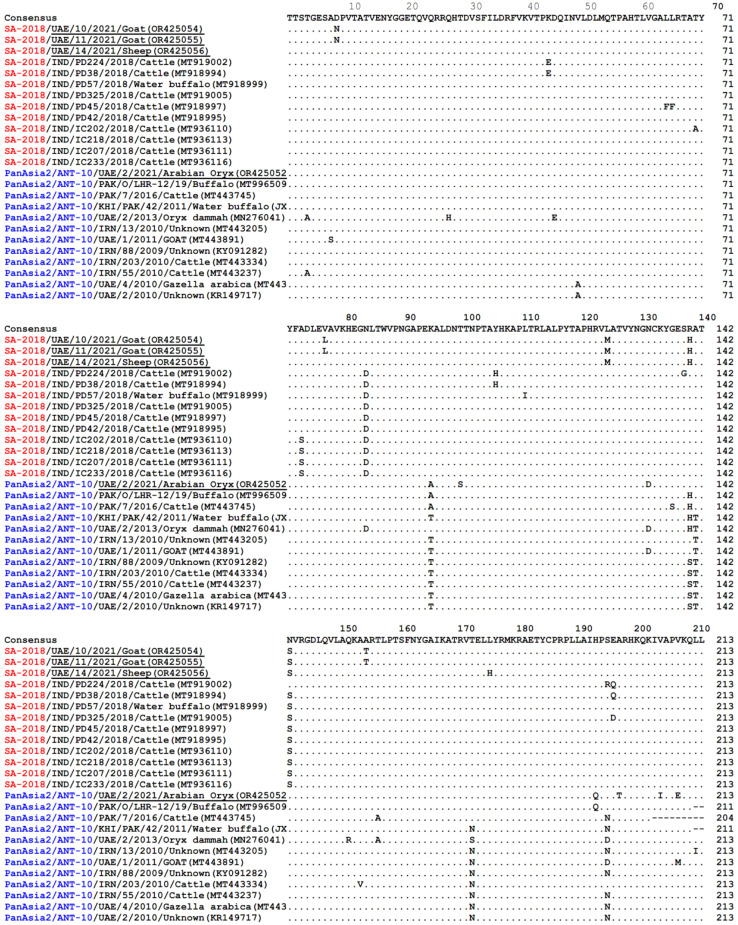
Alignment of VP1 amino acids of FMDV from sheep, goats, and *Arabian oryx*, isolated from UAE (underlined) together with selected reference sequences within the O/ME-SA/SA-2018 lineage and O/ME-SA/PanAsia-2 lineage/ANT-10 sublineage, respectively.

The overall homology in nucleotide sequences between FMDV isolates from the Arabian oryx and isolates from sheep and goats from the UAE was 88%, whereas nucleotide homology among sheep and goat isolates was 98% (Table 3). As expected, the amino acid sequences for O/UAE/2/2021 (O/ME-SA/PanAsia-2/ANT-10) were distinct to those for O/UAE/10/2021, O/UAE/11/2021, and O/UAE/14/2021 (O/ME-SA/SA-2018) (Figure 3). Within the SA-2018 lineage, the UAE samples from goats (OR425054 and 425055) share 98% identity with the UAE samples from sheep (OR425056). Both UAE samples of sheep and goats exhibited 94–97% and 95–98% amino acids with other sequences from India. Within the ANT-10 sublineage, the UAE sample of *Arabian oryx* had 92–97% amino acids identity with different sequences from Pakistan, Iran, or the UAE.

## 4. Discussion

In Middle Eastern countries, including the UAE, FMD is endemic and regularly reported in different animal species. The UAE national animal health plan, which includes FMD, adopts annual mass vaccination of cattle and small ruminants against FMD to control and eradicate the disease. This initiative is in line with the progressive control pathway for FMD (PCP-FMD) of the Global FMD Control and Eradication Strategy and the regional Middle East FMD control roadmap adopted by FAO and WOAH. Principles of the PCP-FMD include active monitoring for FMDV and understanding the epidemiology of FMD. This requires that the strains of FMDV circulating within a country are identified. Within the regional road map, UAE is at stage 2 of the PCP-FMD [13,37].

This study reported six FMD outbreaks that affected *Arabian oryx*, sheep, goats, and cattle located in two different regional locations on five farms and one animal market in the Abu Dhabi Emirate. These outbreaks occurred during April, November, and December 2021, which are considered winter months, providing conditions that may favour viral survival [38]. However, due to the limited sample size, drawing firm conclusions about the influence of climate on disease occurrence in the region requires further investigation.

The infected animals expressed typical FMD clinical signs, including pyrexia, lameness, and vesicular lesions. Although FMD has been previously reported in wildlife, goats, and cattle in UAE [28], this study is the first time that FMD has been described in sheep in the country. 

Three of the FMD-infected farms comprised sheep and goats that had no history of FMD vaccination. Furthermore, the cattle sampled at the animal market were also not vaccinated. Vaccination against FMD is an important tool to control and prevent the occurrence of the disease, especially in endemic regions. Where vaccines are used, it is critically important to reach a high level of vaccination coverage in the targeted livestock population to reduce the incidence of FMD and limit the spread of infection [39]. It should be noted that the target of the vaccination campaign in the UAE was 85%; however, due to COVID-19 lockdown, it was difficult for the veterinary field teams to achieve this target within the planned time. Animal markets in the Abu Dhabi Emirate and elsewhere in the UAE are normally attached to slaughterhouses. Thus, these sites represent a location for gathering and mixing of vaccinated and unvaccinated animals of different species from different sites in the country, as well as for animals imported from other countries. Animals from these markets are either sold for slaughter or transferred to other farms. Thus, animal markets and slaughterhouses may represent a hotspot area for FMD virus occurrence and spread, both from contaminated environments and subclinical infected cattle entering the market [40,41]. Moreover, FMD has a potential zoonotic impact, and it is advised that dairy farmers, laboratory workers, animal handlers, veterinarians, and persons in contact with wild ungulates (zoo workers) take precautionary measures to prevent the disease [4].

The *Arabian oryx* sampled on Farm A were unvaccinated and had been recently moved from captivity where FMD outbreaks were reported. Although there were no clinical signs observed in the other species (sheep and goats) kept on this farm which had received a single dose of FMD vaccine, the movement of such unvaccinated animals has potential to spread the virus after FMD outbreaks [28,42]. One FMD-infected farm (Farm C) contained sheep and goats which had also received a single dose of the FMD vaccine two months before the outbreak. Moreover, this farm was also located within a 5.87 Km radius of the unvaccinated infected farm (Farm B), which may be a risk factor. FMD vaccination, when implemented in combination with effective zoo sanitary measures such as on-farm biosecurity, quarantine, and culling of infected animals, reduces the risk of FMD outbreak occurrence. However, limitations of the commonly used inactivated FMD vaccines include incomplete antigenic matching between the field virus and the vaccine strain, variable antigenic load, antigen instability, cold chain requirements, poor adaptation of some strains to vaccine production, clinically protected animals becoming infected due to nonsterile immunity, high levels of coverage being required to provide protective herd immunity, interference with maternally derived antibodies, short duration of protection, and requirement for repeat boosting. Hence, for adequate protection of sheep and goats against FMD in the Abu Dhabi Emirate, a higher FMD vaccination coverage (up to 100%) with booster doses is recommended [43].

Previous studies in UAE have reported three FMD serotypes (O, A, and Asia 1) [44]. This study confirmed the existence of serotype O and performed molecular analyses to uncover epidemiological links to FMDVs circulating in neighbouring countries [45]. However, the existence of other FMD serotypes in the UAE cannot be excluded due to the very small sample size used in this study. The UAE sequences reported were divergent from the prototype strains of the relevant topotypes by 89–95% and 93–95%, respectively. Such differences are consistent with the continued divergence of these virus lineages within each topotype [46,47]. The UAE sequences detected in sheep and goats were classified within the O/ME-SA/SA-2018 lineage, and this is the first report describing the circulation of this lineage in the region. Moreover, these sequences also clustered separately from other sequences within SA-2018. This indicates the continued divergence of virus lineages within each topotype and the probability of a separate source of origin from the prototype detected in India and Sri Lanka in 2018 and 2019, respectively [46].

Classification of a sequence detected from *Arabian oryx* within the ANT-10 sublineage is not surprising, as this strain was recently detected in 2021 in other Asian countries, including Pakistan, Israel, Palestine, and Jordan [45]. The FMDV detected in *Arabian oryx* in the UAE was most closely related to viruses recently detected in Pakistan, suggestive of a close epidemiological history. This information highlights the potential transboundary transmission pathways to and from neighbouring countries. Thus, the importance of vaccination for the susceptible animals in the farms located at the borders should be prioritised [45]. 

## 5. Conclusions

In summary, this is the first report of FMDV-VP1 characterisation in sheep from the UAE. FMD infection in the UAE remains significant, and this study highlights the connections between FMDV sequences collected in the UAE and the Middle East and neighbouring countries, including Iran, Jordan, Israel, Palestine, and Pakistan. FMD was detected in sheep, goats, and *Arabian oryx* in different locations within the Abu Dhabi Emirate. Two distinct lineages of FMDV, namely SA-2018 and PanAsia-2, were circulating in livestock and *Arabian oryx*, where the former lineage was first detected in sheep and goats in the UAE. These cases occurred in unvaccinated animals, and the virus source was attributed to animal movements, which highlights the importance of increasing vaccination coverage with strict animal movement controls and implementing other zoo sanitary biosecurity measures to reduce opportunities for the virus to circulate.

This study was based on a small number of clinical samples presented to ADAFSA Veterinary Laboratories for routine diagnosis; therefore, it only reflects the presence of the serotype O rather than addressing the wider epidemiological status of the disease across the country and the region. Further systematic epidemiological studies and genomic sequencing data of the virus are required to understand the disease situation in the region, as well as analysis of the risk factors contributing to the transboundary connectivity and spread of the different serotypes in the UAE. Such data are required to enhance the ongoing national animal health control plan, particularly the technical plan for control and eradication of FMD.

## Figures and Tables

**Table 1:** Summary of FMD cases on the farms and market in UAE.

Location of Cases		Notification Date	Livestock Population on the Farm (species)	FMD Affected Animals	Age of FMD Cases	Deaths	Morbidity Rate (%)	Mortality Rate (%)	Case Fatality Rate (%)	FMD Vaccination
Farm A	Abu Dhabi	10 April 2021	132 (sheep 24, Goat 93, *Arabian oryx* 15)	4 *Arabian oryx*	3 years	1 *Arabian oryx*	3	0.76	25	Yes (one dose) ^1^
Farm B	Abu Dhabi	20 April 2021	44 (sheep 27, goats 17)	3 sheep	<3 months	0	6.8	0	0	No
Farm C	Abu Dhabi	21 April 2021	407 (sheep 202, goats 205)	4 sheep	1 year	1 sheep	1	0.24	25	Yes (7 February 2021)
Farm D	Al Ain	18 November 2021	529 ^2^ (sheep 81, goats 448)	94 (80 goats, 14 sheep)	1 year	9 (1 sheep, 8 goats)	0.18	1.70	9.60	No
Farm E	Al Ain	19 December 2021	150 (sheep 100, goats 50)	20 sheep	1.5 years	5 sheep	0.11	0.03	25	No
Market F	Al Ain	1 December 2021	309 (cattle pens only)	11 cattle	4 years	1 cattle	3.56	0.3	9	No

^1^ Fifteen unvaccinated *Arabian oryx* were introduced to the farm three weeks prior to the FMD cases. ^2^ Four unvaccinated goats were introduced to the farm two weeks before the FMD cases.

**Table 2:** Samples collected for laboratory investigation.

Farm Name	Date of Sample Collection	Sample Type	ADAFSA Label	WRLFMD Ref. No.	Animal Species Infected
**A**	April 2021	Mouth swab	ADAFSA-2	UAE/2/2021	*Arabian oryx*
**B**	April 2021	Mouth swab	ADAFSA-9 ADAFSA-10	UAE/3/2021 UAE/4/2021	Sheep
**C**	April 2021	Mouth swab	ADAFSA-11 ADAFSA-12 ADAFSA-13	UAE/7/2021 UAE/5/2021 UAE/6/2021	Sheep
**D**	November 2021	Mouth swab	ADAFSA-4 ADAFSA-5	UAE/10/2021 UAE/11/2021	Goat
**E**	December 2021	Mouth swab	ADAFSA-14	UAE/14/2021	Sheep
**F**	December 2021	Mouth swab	ADAFSA-6 ADAFSA-7 ADAFSA-8	UAE/8/2021 UAE/12/2021 UAE/13/2021	Cattle

**Table 3:** The VP1 nucleotide sequence identity of UAE -FMDV detected in *Arabian oryx*, goat, and sheep.

	O/UAE/2/2021/*Arabian oryx*	O/UAE/10/2021/Goat	O/UAE/11/2021/Goat	O/UAE/14/2021/Sheep
O/UAE/2/2021/*Arabian oryx*		88%	88%	88%
O/UAE/10/2021/Goat	88%		100%	98%
O/UAE/11/2021/Goat	88%	100%		98%
O/UAE/14/2021/Sheep	88%	98%	98%	

## Data Availability

The VP1 gene of the FMDV UAE strains and the sequences generated in this study are available in the NCBI database under the accession numbers mentioned in the manuscript.
